# An agenda for future Social Sciences and Humanities research on energy efficiency: 100 priority research questions

**DOI:** 10.1057/s41599-022-01243-z

**Published:** 2022-06-30

**Authors:** Chris Foulds, Sarah Royston, Thomas Berker, Efi Nakopoulou, Zareen Pervez Bharucha, Rosie Robison, Simone Abram, Branko Ančić, Stathis Arapostathis, Gabriel Badescu, Richard Bull, Jed Cohen, Tessa Dunlop, Niall Dunphy, Claire Dupont, Corinna Fischer, Kirsten Gram-Hanssen, Catherine Grandclément, Eva Heiskanen, Nicola Labanca, Maria Jeliazkova, Helge Jörgens, Margit Keller, Florian Kern, Patrizia Lombardi, Ruth Mourik, Michael Ornetzeder, Peter J. G. Pearson, Harald Rohracher, Marlyne Sahakian, Ramazan Sari, Karina Standal, Lidija Živčič

**Affiliations:** 1 Global Sustainability Institute, Anglia Ruskin University, Cambridge, UK; 2 Department of Interdisciplinary Studies of Culture, Centre for Technology and Society, Norwegian University of Science and Technology, Trondheim, Norway; 3 Department of History and Philosophy of Science, National and Kapodistrian University of Athens, Athens, Greece; 4 Department of Anthropology, and Durham Energy Institute, Durham University, Durham, UK; 5 Institute for Social Research in Zagreb, Zagreb, Croatia; 6 Department of Political Sciences, Babeş-Bolyai University of Cluj, Cluj-Napoca, Romania; 7 School of Architecture, Design and the Built Environment, Nottingham Trent University, Nottingham, UK; 8 Salt River Project Integrated System Planning & Support, Tempe, AZ USA; 9 Unit H1 Knowledge for Policy: Concepts and Methods, European Commission, Directorate-General Joint Research Centre, Ispra, Italy; 10 School of Engineering and Architecture, and Environmental Research Institute, University College Cork, Cork, Ireland; 11 Department of Public Governance and Management, Ghent University, Ghent, Belgium; 12 Sustainable Products and Material Flows Division, Oeko-Institut e.V., Darmstadt, Germany; 13 Department of the Built Environment, Aalborg University Copenhagen, Copenhagen, Denmark; 14 Research Group on Energy, Technology and Society, Électricité de France (EDF), Paris, France; 15 Centre for Consumer Society Research, University of Helsinki, Helsinki, Finland; 16 Independent Researcher, Sesto Calende, Italy; 17 Department of Public Policies and Social Changes, Institute of Philosophy and Sociology, Bulgarian Academy of Sciences, Sofia, Bulgaria; 18 Department of Political Science and Public Policy, Iscte—Instituto Universitário de Lisboa, Lisbon, Portugal; 19 Institute of Social Studies, University of Tartu, Tartu, Estonia; 20 Ecological Economics and Environmental Policy, Institute for Ecological Economy Research, Berlin, Germany; 21 Urban & Regional Inter-university Department, Politecnico di Torino, Turin, Italy; 22 DuneWorks, Eindhoven, The Netherlands; 23 Institute of Technology Assessment, Austrian Academy of Sciences, Vienna, Austria; 24 Centre for Environmental Policy, Imperial College London, London, UK; 25 School of Architecture, Cardiff University, Cardiff, UK; 26 Department of Thematic Studies—Technology and Social Change, Linköping University, Linköping, Sweden; 27 Department of Sociology, University of Geneva, Geneva, Switzerland; 28 Department of Technology, Management and Economics, Technical University of Denmark, Kongens Lyngby, Denmark; 29 CICERO—Center for International Climate Research, Oslo, Norway; 30 Focus Association for Sustainable Development, Ljubljana, Slovenia

**Keywords:** Sociology, Science, technology and society, Geography, Environmental studies, Politics and international relations

## Abstract

Decades of techno-economic energy policymaking and research have meant evidence from the Social Sciences and Humanities (SSH)—including critical reflections on what changing a society’s relation to energy (efficiency) even means—have been underutilised. In particular, (i) the SSH have too often been sidelined and/or narrowly pigeonholed by policymakers, funders, and other decision-makers when driving research agendas, and (ii) the setting of SSH-focused research agendas has not historically embedded inclusive and deliberative processes. The aim of this paper is to address these gaps through the production of a research agenda outlining future SSH research priorities for energy efficiency. A Horizon Scanning exercise was run, which sought to identify 100 priority SSH questions for energy efficiency research. This exercise included 152 researchers with prior SSH expertise on energy efficiency, who together spanned 62 (sub-)disciplines of SSH, 23 countries, and a full range of career stages. The resultant questions were inductively clustered into seven themes as follows: (1) Citizenship, engagement and knowledge exchange in relation to energy efficiency; (2) Energy efficiency in relation to equity, justice, poverty and vulnerability; (3) Energy efficiency in relation to everyday life and practices of energy consumption and production; (4) Framing, defining and measuring energy efficiency; (5) Governance, policy and political issues around energy efficiency; (6) Roles of economic systems, supply chains and financial mechanisms in improving energy efficiency; and (7) The interactions, unintended consequences and rebound effects of energy efficiency interventions. Given the consistent centrality of energy efficiency in policy programmes, this paper highlights that well-developed SSH approaches are ready to be mobilised to contribute to the development, and/or to understand the implications, of energy efficiency measures and governance solutions. Implicitly, it also emphasises the heterogeneity of SSH policy evidence that can be produced. The agenda will be of use for both (1) those new to the energy-SSH field (including policyworkers), for learnings on the capabilities and capacities of energy-SSH, and (2) established energy-SSH researchers, for insights on the collectively held futures of energy-SSH research.

## Introduction

Climate change mitigation represents a critically urgent normative underpinning for research and innovation (e.g., UK Research and Innovation, 2021; United Nations, 2021a). In responding to such a global challenge, there have been increasing calls for research on transitions to low-carbon energy systems that better account for human and societal considerations (e.g., IRENA, 2018; United Nations, 2021b). The first page of the EU’s Green Deal Communication notes that sustainability policy must “put people first” (European Commission, 2019, p. 2).

It is not novel to argue that more Social Sciences and Humanities (SSH) research would help drive low-carbon energy transitions. The research literature shows that involving SSH can ensure insights on, for example, the societal influences, relationships, preconditions, interventions, outcomes, impacts, etc. of energy transitions (Foulds and Robison, 2018; Ingeborgrud et al., 2020; Robison and Foulds, 2019; Sovacool, 2014; Sovacool et al., 2015). There is a clear energy policy need for SSH evidence, which decades of techno-economic policymaking have often struggled to deliver. It is therefore welcome that (policy-focused) research funders are responding to this urgent need. For example, both of the EU’s most recent Framework Programmes (Horizon 2020, 2014–2020; Horizon Europe, 2021–2027) contain commitments for SSH to be ‘mainstreamed’ across their collective €174.5bn of project investments (European Commission, 2021a).

We note two main sets of gaps in the delivery of more and better interdisciplinary SSH research on low-carbon energy transitions within Global North contexts. The first set of gaps relates to how the SSH input sought by policy and funders (usually under the banner of generating policy evidence/recommendations) is a poor representation of SSH and what these can offer (Foulds and Christensen, 2016; Genus et al., 2021). For example, SSH are often deployed to support pre-determined, top-down policy initiatives, as part of seeking to persuade citizens and ensure ‘acceptance’ (Robison and Foulds, 2021) and usually in a subordinate role to the Technical/Natural Sciences (Kropp, 2021; Silvast and Foulds, 2022). Indeed, SSH research communities are very rarely involved in the setting of funding agendas, which ironically they are then expected to be integral deliverers of (Royston and Foulds, 2019). The alienation of SSH research communities from setting major funding programmes and policy processes is commonplace and systemic—with decision-makers often being ambivalent about SSH (Pedersen, 2016)—as demonstrated by SSH actually only receiving a small fraction of the research funding available. For example, Kania and Bucksch (2020) noted that SSH partners only received 10% of funding in the energy work programme of Horizon 2020 in 2018; and Overland and Sovacool (2020) showed that only 0.12% of funding on climate mitigation research more widely (from 333 funders, over 1990–2018) was spent on the Social Sciences. All this matters because funding systems (through, e.g., evaluation protocols, call wordings, etc.) actively co-construct the range of energy research findings and policy recommendations possible (Royston and Foulds, 2021; Shove and Rip, 2000; Silvast and Foulds, 2022). Furthermore, SSH is of course not a homogenous category. In an empirical study, Royston and Foulds (2019) found that the SSH informing EU energy policy was strongly dominated by a single discipline: Economics, with the interpretive SSH playing a very small role. There is a need not only for a greater use of SSH in general, but also for a wider variety of SSH approaches to be used in energy research/policymaking.

The second set of gaps concerns a lack of deliberative processes that bring together energy-SSH researchers from different disciplines to frankly discuss, gap-spot, negotiate, prioritise, craft and even ‘dream up’ research agendas. Although SSH researchers are obviously usually involved in the implementation stages of interdisciplinary SSH projects, there remain few systematically structured and well-resourced initiatives that provide opportunities for SSH researchers to actually engage in deliberative agenda-setting work. This is to the detriment of advancing knowledge generation across energy-SSH research communities. Instead, future ‘research agendas’ are commonly proposed by colleagues through, for example: offering individual perspectives based on their own experiences and reflections (e.g., Henning, 2015; Royston et al., 2018); relying on past literature, which inevitably creates less room for blue-skies thinking and forward-looking possibilities (e.g., Andrews and Johnson, 2016; Martiskainen and Sovacool, 2021); and/or, self-limiting themselves within their community’s own disciplinary boundaries (e.g., Steg and Vlek, 2009). Many energy-SSH research agendas also fail to transparently detail how exactly the agenda was formulated (e.g., Steg et al., 2021)^1^. We are not asserting that these contributions are not useful in shaping the directions of travel for researchers, funders and policy-research systems. However, we do argue that such studies are unlikely to provide an inclusive and comprehensive agenda for research, simply because they are not systematically negotiated with peers and rarely seek out different perspectives. In other words, they are not deliberative approaches to agenda-setting. Indeed, the pragmatic outcomes associated with embracing wider processes of negotiation (e.g., cross-fertilisation, peer learning, shared ownership) are what provide the credibility and long-term legacy of any output (e.g., published research agenda). Although there are some emerging examples of good practice that aim to ‘co-create’ energy-related SSH research agendas (e.g., Köhler et al., 2019; Sovacool et al., 2020), the academy needs to go further.

Horizon Scanning is a method that explicitly aims to inform emerging research agendas, through a systematic, negotiated process undertaken by carefully selected groups of scholars. The method (or more appropriately, set of methods) has been used to inform emerging research priorities in a number of sustainability-related fields, including ecology and biodiversity conservation (Sutherland et al., 2019), sustainable agriculture (Bharucha et al., 2021a; Pretty et al., 2010), pharmaceuticals and the environment (Boxall et al., 2012), and water (Brown et al., 2010). These exercises aim to canvass the thoughts of a wide, cross-disciplinary field of scholars, using their expertise and unique perspectives on their own discipline(s) to ‘look forward’, in identifying important knowledge gaps or priorities for further funding, policy attention, and/or research. The typical approach is to convene a ‘core group’ of experts who in turn consult their own networks, and then undertake a structured, iterative process of deliberation to arrive at a final list of questions considered to be priority directions for the discipline(s). However, despite such promise, Horizon Scanning has not yet been utilised for co-creating energy-SSH research agendas.

Inspired by such methods and aforementioned gaps, this paper aims to set out a collectively elaborated research agenda outlining future SSH research priorities for energy efficiency. This is an SSH research agenda; designed by SSH researchers, to be implemented by SSH researchers, and with clear interdisciplinary relevance across a range of SSH disciplines. It is important to note that this present paper is not concerned with enabling greater interdisciplinarity between Science, Technology, Engineering and Mathematics (STEM) and SSH communities working on energy, or supporting mainstreaming of SSH into STEM. These are important issues that have been extensively covered elsewhere (e.g., Cooper, 2017). Instead, our paper digs more deeply into the energy-SSH field, intentionally spanning across its rich array of divergent ontologies. Indeed, the purpose of our Horizon Scanning exercise (as per others such as, e.g., Pretty et al., 2010, Bharucha et al., 2021b; Orr et al., 2022) is to systematically identify research gaps and novel areas for further enquiry. The primary audience for this paper is thus energy-SSH researchers. It is also important to note that our contribution to this audience is an empirically derived overview of a diverse field, rather than a (relatively narrow) theory-driven perspective on particular kinds of SSH engagement. Indeed, such a theoretically driven engagement would run counter to the basic logic of Horizon Scanning, which, as the term suggests, is designed to offer a broad overview of diverse fields and fast-evolving challenges.

While we made the exercise more tangible for our participants by focusing on EU Horizon Europe’s future priorities^2^, we do not believe the value of this agenda should be limited only to shaping EU research directions. The questions generated are of relevance to a range of Global North contexts, as suggested by their resonances with literature based in the USA and Australia, among other settings. Regrettably, our exercise did not have scope to consider the vast and important topic of energy efficiency in Global South contexts.

Our Horizon Scanning exercise sought to identify 100 priority SSH research questions, which were inductively clustered into relevant themes. This exercise aimed to be inclusive across the full range of SSH disciplinary contributions—as well as, e.g., geographies, nationalities, career stages, academic/industry contexts—and intentionally showcases the variety of different SSH possibilities (e.g., for policy evidence) that can be produced. We anticipate that our agenda (i.e., the 100 priority questions and their associated themes) will be of use for both (1) those new to the energy-SSH field, for learnings on the capabilities and capacities of energy-SSH, and (2) established energy-SSH researchers, for insights on the collectively held futures of energy-SSH research.

Energy efficiency represents an interesting focus for SSH-centred Horizon Scanning, given that it is itself a techno-economic concept that has been adopted by policymakers, and is an area in which SSH have much to offer (in terms of, e.g., delivery, critiques and alternatives) yet are still usually overlooked (Dunlop, 2019; Guy and Shove, 2000). Energy efficiency has been a mainstay of energy policy in recent decades and this does not look set to end (Dupont, 2020; Saunders et al., 2021); for example, the EC’s 2021 Fit for 55 package proposes to formally embed its ‘Energy Efficiency first’ principle as a legal provision (European Commission, 2021b); while the International Energy Agency (IEA) describes energy efficiency as “The first fuel of a sustainable global energy system” (International Energy Agency, 2021 no pagination). Within this paper, we utilise the European Commission’s (2012) Energy Efficiency Directive’s broad definition—“energy efficiency means the ratio of output of performance, service, goods or energy, to input of energy” (article 2, point 4; p.10)—to ensure our scope of enquiry regarding (increased) energy efficiency remains policy relevant.

This paper proceeds as follows: we begin by discussing the recent evolution of SSH research on energy efficiency. We then present our Horizon Scanning approach, including the ways in which participants were involved in shaping and co-owning the final Horizon Scan agenda. The core of the paper then presents our 100 SSH priority questions, grouped into seven themes. We conclude by reflecting on: what our agenda offers and how it may be used; what it indicates about past/future directions of SSH research on energy efficiency; and, what our e.g., inductive, inclusive, and pragmatic approach has demonstrated to the field.

## Background context: the evolution of Social Sciences and Humanities (SSH) research on energy efficiency

The story of SSH research on energy efficiency has a long history. For example, in the latter half of the 1800s: William Stanley Jevons conceptualised the rebound effect (Jevons, 1865); and Podolinsky explored ‘Energy Return On Investment’ (EROI) in relation to human labour and activities, and thus was regarded as pioneering ‘social energetics’ (Martinez-Alier, 1987, pp. 45–63). Early Humanities studies also engaged in these debates, such as when: Middle Ages Historian, Marc Bloch, discussed the lack of energy system development due to slave labour (Bloch, 1935); and, Anthropologist, Leslie White, described the role of energy in cultural evolution (White, 1943).

Much of these early commentaries involved an emphasis on how technological progress and steeply rising energy demand were widely taken for granted. This changed dramatically in the wake of the oil crises of the 1970s when first political, and then environmental, vulnerabilities of depending too much on (foreign) oil became a political priority. Fuel scarcity and high oil prices forced oil-importing governments to ration access, impacting on productivity. Therefore, after the fall of naive visions of continued progress in the ‘atomic era’ (Nelson, 2014), energy efficiency entered the scene as a quick fix to preserve a high-energy way of life. However, in the following years, it became clear that the wide adoption of energy efficient solutions required culture and society (which fundamentally relied on cheap energy) to change.

Confronted with the explosive growth of energy consumption and resulting crises, SSH research in the 1980s focused on understanding human behaviours related to energy use, and how such behaviours are grounded in specific environments. This was also the starting point of a report by the USA’s National Research Council, entitled ‘Energy use: the human dimension’ (Aronsen and Stern, 1984). This report, which acted as a landmark in energy-SSH far beyond the US context, outlined central themes that have remained relevant to research on energy efficiency, including: energy’s ‘invisibility’ to its users; problems of energy information; the symbolic meanings of energy use; household energy interventions; the role of intermediaries in energy use; social equity; and local energy action.

Perhaps the most significant shift in energy-SSH research since the 1980s has been a move away from a focus on preparedness for energy security. Instead, energy efficiency became more and more framed as part of broader responses to another crisis: climate change (e.g., Geels et al., 2018), with much current work on energy efficiency now framed in terms of low-carbon transitions or transformations, and the role of niches, regimes and different kinds of innovation (both technical and social) within these systemic shifts (Lieu et al., 2020; Wittmayer et al., 2020). Another shift, of course, is the continuing development of SSH research that evaluates and assesses policies and regulations, as these policies themselves evolve (e.g., Bergman and Foxon, 2020; Dupont, 2020; Kern et al., 2017; Lovell, 2004; Lutzenhiser, 2014).

However, there have also been some strong continuities in energy-SSH since the 1980s, and much of the USA’s National Research Council report reads like a research programme that was implemented in the following decades. For instance, what Aronsen and Stern (1984, pp. 161–181) call ‘local energy action’, has been extensively studied as energy community initiatives (Catney et al., 2013; Hielscher et al., 2011). Moreover, with the persistence of social and economic inequalities at various scales and the roll-out of climate change mitigation measures, SSH research has continued to focus on how energy and social justice relate to each other, with attention paid to, for example, gendered differences in the management of residential energy efficiency (Carlsson-Kanyama and Lindén, 2007; Standal et al., 2020) and the links between energy efficiency and fuel poverty (Gillard et al., 2017).

Another research area, which has developed from pioneering work in the 1980s, concerns household-level interventions and their efficacy (for an overview, see McAndrew et al., 2021). Analyses of rebound effects and unintended consequences—from macroeconomic modelling to household-level studies (Gram-Hanssen et al., 2012; Winther and Wilhite, 2015)—have developed Aronsen and Stern’s basic argument that energy efficiency improvements need to account for the contexts of everyday life, or else will face problems of ‘user behaviour’. More recently, research grounded in the critical-SSH has widened debates on energy efficiency by exploring emotional geographies, lived experiences and sensory ethnographies of energy consumption, and by approaching energy technologies as elements of material cultures (Chappells and Shin, 2021; Shove et al., 2014).

This work on household dynamics, materiality and daily life intersects with a wider body of literature that originated in the 1980s (e.g., Wilk and Wilhite, 1985), and has burgeoned from the early 2000s. This literature argued that mainstream efforts to incorporate SSH insights into energy policy often involved a simplified and instrumental approach. In contrast, this innovation in SSH research on energy and energy efficiency aimed to develop:

“a new approach to the science of energy demand: one which adequately accounts for the actors, institutions and networks which contribute to change; which re-envisions the object of enquiry as the services which energy provides; and which is equipped to understand change.” (Wilhite et al., 2003, p. 123)

Through enlisting conceptual resources on Theories of Social Practice mainly from Schatzki (2002,1996) and Reckwitz (2002)—and thus further building on earlier developments (e.g., Bourdieu, 1977; Giddens, 1984)—these authors have rejected the idea that policy could/should focus on influencing individuals’ choices. Instead, they ask critical questions about what should be achieved by SSH research on energy. This new approach explicitly aims to move beyond the polarisations of technological determinism and social determinism, and also between individual agency and social structure (Shove et al., 2012). In relation to energy efficiency, the most important contribution here has been the problematisation of energy efficiency as an unequivocal goal (Shove, 2018). For instance, some work within this school focuses instead on sufficiency, asking not how to achieve an arbitrary goal with less energy input, but what it is that should be achieved with energy—towards conceptualising the good life (Darby and Fawcett, 2018; Princen, 2003).

Unsurprisingly then, SSH research on energy in general and specifically on energy efficiency has developed with the changing role of energy in society. As long as energy appeared as unproblematic, it was very often ignored by SSH researchers. This changed dramatically when access to abundant energy, enabled by cheap fossil fuels, became deeply problematic. As energy resources have appeared more and more precarious, new SSH disciplines have added their respective approaches and bodies of knowledge, creating a heterogeneous group, which addresses energy efficiency from different angles. Today, SSH researchers also help to increase energy efficiency by studying what people and institutions know and do—including those who are tasked with implementing energy efficiency (Cooper, 2018)—as well as reflecting critically on SSH’s ability to engage with energy as a technical entity and exploring what changing a society’s relationship to energy means in more fundamental terms (Love and Cooper, 2015; Cooper, 2017).

As we look forward, it is clear that past SSH research on energy efficiency provides a rich body of knowledge. Well-developed approaches, ranging from practical support for efficiency interventions to fundamental critiques, are ready to be mobilised to contribute to the generation of what has often been called ‘the greenest form of energy’: energy, which is not used. As the role of energy in society continues to change, new challenges arise. Energy efficiency’s unique promise—to do more with less—is an offer with considerable traction among energy policymakers, not least because it fits within the dominant paradigm of continual economic growth. It is therefore reasonable to assume that societies will continue to commit to energy efficiency interventions in tackling the challenges of the coming decades; SSH research on energy efficiency must inevitably form part of this.

## Methodology: how Horizon Scanning produced 100 research questions

This section details how we conducted our Horizon Scanning exercise. Our question selection approach was inspired by Sutherland et al.’s (2019) similar annual biodiversity conservation Horizon Scanning exercises. In detailing our adaptations and processes, we particularly respond to a lack of methodological specificity in most Horizon Scanning literature. An even more detailed preliminary methodological plan is presented elsewhere, in the context of the wider Horizon Scanning literature (Foulds et al., 2019a).

First, a Steering Committee was formed (the first four co-authors of this paper), whose responsibility it was to oversee the Horizon Scanning exercise^3^. This Committee began by writing its Terms of Reference (Foulds et al., 2019b), which outlined the boundaries/scope (e.g., energy efficiency definition) and policy contexts of operation for our Horizon Scanning. The Steering Committee was supported in producing the Horizon Scan by a Working Group (latter 27 co-authors), whose responsibility it was to gain wider input from their research communities and to help select and edit the final 100 questions and themes^4^. The Group’s members all held a researcher identity (whether in academia or industry), were based in organisations/countries eligible for EU Framework Programme funding, and held SSH research interests directly relevant to energy efficiency. The members spanned 21 different countries, with 11% of members working in non-EU Horizon 2020 Associated countries^5^. The countries had a regional breakdown^6^ of: 33% in Northern Europe and other surrounding Northern (e.g., non-EU) countries; 11% in Eastern; 26% Southern; and 30% Western. They were 52% women and 48% men, and represented 29 different SSH (sub-)disciplines, with 26% participants having prior Science, Technology, Engineering and Mathematics (STEM) backgrounds. Further, 33% were classified as ‘frontrunners’ and 67% as ‘field leaders’^7^. Originally, there were 31 members, but four dropped out mainly because of our timelines coinciding with the first waves of the COVID-19 pandemic. Fuller information on recruitment selection criteria and targets for Working Group members are available in Foulds et al. (2019a, pp. 17–18).

The Steering Committee and Working Group then sought input from wider research communities, with each member invited to submit their own questions and invite up to 20–25 colleagues for input. Input was gathered via an online survey (February–April 2020), which asked for 3–5 SSH research questions on energy efficiency, each with an accompanying justification. Eligibility criteria required these colleagues to have prior SSH expertise on energy efficiency and be currently based at a research organisation in an EU or Horizon 2020 Associated Country. Our intention behind our Working Group involving a cross-section of SSH disciplines, genders and European geographies, was to ensure a degree of balance to the wider pool of submitted questions. We recognise that the definition of our geographic scope, as EU and Associated Countries, is a limitation of this exercise, and that deliberative exercises encompassing wider scales would be valuable in expanding the inclusivity of SSH research agendas. As with all Horizon Scanning exercises, our dataset is thus highly contingent on the precise group of participants recruited. As noted elsewhere in the Horizon Scanning literature (e.g., Orr et al., 2022), a different set of participants would have resulted in a different set of questions. It is worth highlighting though, that this is not necessarily a weakness of the methodology, for two reasons. First and more generally, all qualitative, deliberative methodologies share this feature of data being contingent on participants’ unique perspectives, positions and experiences. Second, in attempting to systematically canvas as *diverse* a field as possible, we have made every reasonable attempt to gain as representative a sample of participants as possible within the practical confines of a single research study.

Survey respondents totalled 152 (62% men, 37% women, 1% rather not say; 78% had completed a Ph.D), and they spanned 62 (sub-)disciplines of SSH. Economics was the most represented, with 23% of respondents stating this as one or more of the SSH they aligned with; Sociology had 21%, Sciences and Technology Studies and Political Science (each had 13%), and Human Geography (11%). The respondents also spanned 23 countries and 26 nationalities, and represented a full range of career stages; a breakdown of these is available in Foulds et al. (2020 p. 27). These survey responses yielded 513 submitted research questions. This is the exercise’s core source data, and has been anonymised and made available by the Energy-SHIFTS Consortium (2021). Thus, what comes next in our methodology is the cleaning, sorting and editing of these submitted questions.

The purpose of this next stage was to produce a list of questions that were consistent in their writing style, relevance, scale and scope. The first and second authors of this paper took responsibility for this editing task, with a number of inter-editor agreement checks to ensure robustness (c.f. Campbell et al., 2013; Nowell et al., 2017). For example, three such checks involved: a pre-meeting agreed the editing rules (inspired by the criteria used by Sutherland et al. (2011) and Pretty et al. (2010) on what constitutes good research questions); before working independently, 5% of the submitted questions were randomly chosen and edited by *both* editors separately, with an agreement meeting to discuss the processes/outcomes and to jointly agree on final edits; and, another 5% were similarly jointly edited at the halfway point. This task led to:

- 101 submitted questions deleted, due to lack of SSH grounding, lack of relevance to energy efficiency, or not containing question content.
- 35 additional questions added through disaggregating multiple questions from one single submitted question, or through sourcing questions from justification texts.
- 64 submitted questions removed due to merging, as they were overly similar.

This produced a list of 383 edited questions, which the Steering Committee and Working Group evaluated (June–August 2020) individually, with all questions scored on a scale of 1 (=definitely exclude) to 5 (=definitely include). Participants were asked to score each question based on how important they believed it should be for the European Commission to consider it within its Horizon Europe funding programmes. At this stage, rather than using an elaborate (and potentially quite constraining) set of pre-determined criteria, scorers were instructed to assign scores based on their first, strong preference, grounded in their expertise and unique disciplinary vantage points. There were no restrictions on the number of questions you could, for example, score highly.

The results produced: a list of 50 selected edited questions; a long-list of 150 edited questions that had potential for selection; and a rejected list of 183 questions that were scored too poorly to be included. There was a clear pattern in more conventional questions being less popular in the evaluations (e.g., those associated with Economics and Business Management—especially including the very many questions relating to the rebound effect). The rules applied were:

- Questions with a median of 5 were automatically selected for inclusion. (*n* = 1)
- Highest-scoring 49 questions were selected^8^, to create a shortlist of 50. (*n* = 49)
- Questions with medians of 1–3 were automatically excluded. (*n* = 183)
- Remaining edited questions compiled as a long-list for Working Group consideration. (*n* = 150)

All evaluation scores and rulings were transparently shared with the Working Group. Following this, a first virtual deliberation workshop discussed the results with Working Group members. Discussion was prompted by each member advocating for three questions from the 150-question long-list. We gathered feedback on specific questions that should be included, with agreement reached that not enough of the selected questions to date had covered, e.g., gender, the Global South, social innovation, and unintended consequences. Here too, rather than using a pre-determined set of criteria against which to judge all questions, we followed a reflective and deliberative approach, which allowed Working Group members to holistically consider each question on its own merits and against the overall goal of producing a list of 100 questions that adequately captured the breadth of SSH approaches to energy efficiency.

Following this first workshop, the Committee provisionally selected 95 questions as well as inductively generated seven themes to organise said questions around. A second virtual deliberation workshop discussed the: salient gaps that the final five questions needed to fill; theme descriptions; overall mission statement; and associated narratives accompanying the final 100 questions. Both workshops involved members making direct text edit suggestions to the selected questions. The final 100 selected questions were signed off by members prior to publication.

## Horizon Scan findings and discussion: 100 Social Sciences and Humanities (SSH) priority questions across seven themes

We produced a Mission Statement, which represented the Working Group’s shared intention behind these 100 questions and seven themes. This Statement was produced after the questions had been agreed and the themes inductively generated:

To promote SSH research that better situates energy efficiency in relation to social systems of energy demand and supply; and to constructively challenge notions of energy efficiency by opening up questions of its meanings, applications and implications across diverse contexts, actors and scales.

This section presents the 100 questions, broken down under each of our seven themes. In these seven sub-sections that follow, we discuss each theme in terms of synergies between recent calls in the literature and our own advocated lines of SSH development.

While further information on how to navigate these questions is available elsewhere, including how it is out-of-scope to discuss herein how these questions should be investigated (Foulds et al., 2020, pp. 7–8), we do note that these questions/themes are not intended to be comprehensive and their ordering does not indicate any forms of prioritisation. We also note that our set of 100 questions was produced alongside: an accompanying annotated bibliography, which contextualises our questions in line with seminal SSH work on energy efficiency (Foulds et al., 2021); as well as three other sister Horizon Scanning exercises, which similarly selected 100 priority questions for other energy policy areas^9^.

### Theme 1: Citizenship, engagement and knowledge exchange in relation to energy efficiency

This theme focuses on ideas around engagement, participation and the sharing of knowledge in relation to energy efficiency (Table 1). Implicitly, this theme aligns with calls to move beyond narrow, individualistic forms of participation (Pallett et al., 2019), such as, e.g., information provision. For instance, many of this theme’s questions embrace a fuller, systemic range of forms of participation, such as, e.g., grassroot NGOs, alternative community-based business models, and citizen-focused participatory policymaking initiatives. Overall, the aim of this theme is to investigate practical issues of designing and implementing interventions for engagement and participation, but also to explore deeper issues of what constitutes meaningful engagement, and how engagement initiatives might go beyond a focus on individual behaviour change.

**Table 1:** Theme 1 Social Sciences and Humanities (SSH) priority questions.

Question no.	Agreed SSH priority question
1	How can the development and implementation of energy efficiency measures be democratised; in particular, how can policy choices around energy efficiency technologies be discussed and enacted through inclusive citizen participation?
2	What is the role of ground-level associations (e.g., community companies, trusts, charities, and other kinds of non-governmental organisations) in shaping and achieving energy efficiency goals; and how can localised approaches support citizens’ active participation in energy systems?
3	To what extent are local energy initiatives currently active in the field of energy efficiency and sufficiency; what business models and practices are they employing; and how can these existing local initiatives be scaled up?
4	What social and procedural components need to be considered in establishing community-based energy efficiency projects; and how can these considerations be most effectively incorporated into project design and implementation?
5	How can ‘real laboratories’—such as urban experiments that co-design, carry out, observe and evaluate complex social change processes—contribute to energy transitions?
6	What are the challenges to mobilising collective action around energy efficiency and sufficiency (e.g., convincing private apartment owners to undertake collective refurbishments); and what learnings on addressing these can be drawn from exemplars?
7	What forms of resistance emerge in response to energy efficiency measures; and what is the impact of negative narratives (e.g., conspiracy theories) around these measures?
8	Which organisations and individuals play important roles in the diffusion of energy efficiency measures to homes and businesses; and how do these diffusion processes operate (e.g., through developments in leadership, social norms and skills)?
9	What constitutes meaningful and long-lasting citizen engagement in energy efficiency policy; how can it be enabled and replicated; and who should or could participate in co-creating energy efficiency policies and actions?
10	What purposes are pursued by citizen engagement for energy efficiency (e.g., improving democracy, fostering a low-carbon transition); what synergies and conflicts exist between these purposes; and to what extent does citizen engagement achieve these goals?
11	Who is being constructed as the target of energy efficiency policy (e.g., citizens, consumers, businesses); and how does this construction vary among governance actors?
12	What different kinds of social learning and participatory engagement, among which social actors, are needed in order to address energy efficiency/sufficiency challenges and scale up innovative energy efficiency solutions?
13	How can policies and public programmes aiming to increase energy efficiency, via citizen engagement, go beyond individualistic models of behaviour; and how can a social practice framing result in different types of programmes?
14	What are the relationships, if any, between well-being and citizen participation on energy efficiency/sufficiency issues; how do the different ways of engaging people affect well-being; and what different participatory methods can be experimented with?

A central thread across these 14 questions is how they collectively move beyond the dominance of ‘energy consumers’ within past energy-SSH, to consider ‘energy citizens’. This move aligns too with many recent calls in the literature to better recognise the active role that communities can and should play at the heart of a democratic energy system (e.g., Olawuyi, 2021; Ruostetsaari, 2020). Such a position is markedly different from maintaining a focus on energy consumers, who would tend to represent passive, market customers at the end-point of the energy system (Ryghaug et al., 2018). In conjunction with this appreciation of energy citizenship, the questions also demonstrate a shift from centralised, one-size-fits-all approaches, to more localised, immersive approaches that hope to better understand how collective action can be mobilised (Turcu et al., 2014). This move towards energy citizens is also apparent in the evolution of energy policy texts; although, on closer inspection ‘energy citizens’ are still very often regarded as passive energy end-users (c.f. Lennon et al., 2020)—SSH research has the capacity to drive meaningful change here.

While citizens and communities are central, this theme also encompasses engagement and knowledge exchange in relation to other social actors. The breadth in this theme is also evident in these contexts, specifically with regard to the forms (and implicit conceptualisations) of social innovation that may be effectively utilised (Wittmayer et al., 2020). Although often remaining implicit within the questions, such social innovations prioritised are those relating to (social) learning, cross-sectoral stakeholder interactions, and participatory/co-creation methods. This theme therefore takes us away from unidirectional knowledge transfer, to multidirectional knowledge exchange, where all stakeholders have an active voice and role.

All in all, prioritising matters of citizenship and multi-stakeholder participation will help SSH research on energy efficiency to move beyond narrowly instrumental approaches to user uptake or acceptance. Fundamentally, this theme warns against falling into the well-trodden path of adopting SSH as a tool for convincing/persuading people to accept pre-determined, top-down (usually techno-economic) policy solutions (Robison and Foulds, 2021; Stilgoe and Cohen, 2021). Instead, this theme makes clear the value of research into the generation of ideas, interventions and cross-fertilisation via bottom-up, localised and participatory means.

### Theme 2: Energy efficiency in relation to equity, justice, poverty and vulnerability

This theme centres on the relationships between energy efficiency and various forms of equity, justice, poverty and vulnerability (Table 2). The plurality here is a critical aspect of this theme, in that it emphasises the many forms, spaces and scales through which the impacts of energy efficiency are felt. The management of these impacts is only made more complex, given that the experiences of impacts are unevenly distributed across societies (Wamburu et al., 2021). The core agenda of this theme is therefore to investigate how energy efficiency policies and interventions intersect with these issues, and how their positive effects can be optimised and negative effects minimised.

**Table 2:** Theme 2 Social Sciences and Humanities (SSH) priority questions.

Question no.	Agreed SSH priority question
15	What are the major barriers and enablers of installing energy efficiency measures among different socio-demographic and socio-economic groups; and what are the implications for designing policies that ensure energy efficiency is accessible to all?
16	To what extent can intersectional insights—regarding a person’s social identities (e.g., gender, race, class, sexuality, religion, disability, etc.)—inform the design of energy efficiency solutions, and assist in devising strategies that address their unintended consequences?
17	What are the links between energy efficiency and energy justice at regional, national and global scales; and how can distributional impacts of energy efficiency policies be meaningfully evaluated and fairly managed across societies?
18	To what extent do existing energy efficiency policies, tools and initiatives employ a social justice approach; what would be the implications of embedding a social justice approach throughout policymaking on energy efficiency; and how can this best be achieved?
19	How does a fair distribution of energy efficiency costs and benefits feature in societies’ idea of acceptability; including, what exactly does ‘fair’ mean to different stakeholders?
20	What role can be played by niche or innovative technologies, and by niche innovation management, as mechanisms to secure wider distribution of power, democratic engagement and more just transition management?
21	How do energy efficiency improvements affect inequalities (including across, e.g., socio-economic groups and genders); and how can policies be designed to achieve both energy efficiency and equity goals?
22	How can energy efficiency be increased without increasing energy inequality; in particular, how can the allocation of EU funds take account of the different forms of energy services deprivation that exist across, and within, European countries?
23	What roles do material culture and interactions with technologies play in shaping the distributional inequalities experienced through different energy efficiency initiatives?
24	How do energy efficiency policies affect vulnerable groups with higher energy consumption needs (e.g., elderly, disabled); and how can policies ensure that such ‘energy vulnerable’ citizens benefit from energy efficiency solutions?
25	What kinds of institutional innovations are needed to ensure that energy efficiency policies serve to redress, not exacerbate, energy vulnerabilities; and what lessons can be learned from existing good practice in this area?
26	How significant is energy efficiency in alleviating existing energy poverty across different countries; and how can affordable energy efficiency programmes be supported, as part of delivering fairer energy futures?
27	To what extent do (i) current levels of poverty, including energy poverty, (ii) structure and quality of jobs, and (iii) inequalities within different countries, impact on the capacity for and actual delivery of energy efficiency improvements; and how do these vary across different countries?
28	How can energy efficiency be effectively embedded in future policies targeting energy poverty, and poverty alleviation more generally; and how can such policies be informed by more holistic, interdisciplinary and cross-sectoral approaches?
29	What are the short-, medium- and long-term effects of domestic energy efficiency improvements on the mental and physical health of people living in energy poverty?
30	How might ‘efficiency’ as a conceptual approach exacerbate vulnerabilities; and how can a sufficiency approach support just energy transitions?

As such, the questions closely align with calls from the relatively young energy justice literature, which has already included some direct applications to energy efficiency (e.g., Brooks and Davoudi, 2014; Xu and Chen, 2019). Within the energy justice literature, there has been much attention in recent years on three tenets: distributional justice, i.e., sharing of impacts; recognition justice, i.e., acknowledging social inequalities; and procedural justice, i.e., participating in decision-making (Jenkins et al., 2016; McCauley et al., 2013). While procedural issues around participation were central to Theme 1 (as described above), many of Theme 2’s questions here prioritise: distributional justice, in that they advocate for better understandings of who is impacted, what exactly that impact is, and how it is experienced; and recognition justice, in that they call for institutional acknowledgement of those who are marginalised and vulnerable. Regardless though, these questions do build on an active tradition within energy justice literature of challenging policy approaches and proposing recommendations (e.g., Sovacool and Dworkin, 2015).

It is important to note that all of this theme’s questions should be understood as relating to people’s multiple and intersecting characteristics and vulnerabilities. During the deliberative workshops, the Working Group especially highlighted the role of gender as a cross-cutting issue for this theme, but were also vocal on many other important characteristics that needed consideration, including (but not limited to): age, income, ethnicity, disability, family structure and religion. This certainly fits with the literature’s small, but growing, appreciation of intersectional issues in energy efficiency (e.g., Lewis et al., 2020). Relatedly, this theme reiterates the importance of explicitly confronting the interconnections between different forms of vulnerabilities, including how they may reinforce and exacerbate one another (e.g., those already classified as vulnerable or in poverty, versus those classified as being specifically energy vulnerable or in energy poverty).

### Theme 3: Energy efficiency in relation to everyday life and practices of energy consumption and production

This theme centres on the relationships between energy efficiency, energy demand and people’s everyday lives, including practices of consumption and production, and the wider systems of provision in which these are embedded (Table 3). The aim of this theme is to explore how energy efficiency policies and interventions intersect with ordinary practices and lived experiences, including how everyday life is structured, for example, in time (Shove, 2009), and differentiated, for example, by gender (Anfinsen and Heidenreich, 2017).

**Table 3:** Theme 3 Social Sciences and Humanities (SSH) priority questions.

Question no.	Agreed SSH priority question
31	How do energy efficiency policies (e.g., energy pricing policies) affect everyday life for different groups, especially vulnerable groups and different gender identities?
32	How do different households and social groups understand energy efficiency and energy sufficiency in relation to their everyday lives and practices?
33	What are the emerging (disruptive) energy efficiency technologies that might significantly transform the ways people live and work?
34	What are the relationships between widespread uptake of energy efficiency improvements and changes in social practices of production and consumption?
35	How do new sociotechnical configurations of energy generation, and evolving systems of provision, relate to energy efficiency programmes; for example, what, if any, are the consequences of community-based energy schemes?
36	How do new technological energy efficiency measures interact with practices and infrastructures in consumers’ everyday lives; and how are citizen values, relationships, and institutions reshaped by these technological changes?
37	What unanticipated challenges and poor outcomes arise from a lack of ‘fit’ between new initiatives or technologies with everyday lives and practices; and how can these be addressed?
38	What are the roles of personal, cultural and site-specific factors in the success or failure of energy efficiency initiatives?
39	How can participatory design and co-creation approaches contribute to the development of energy efficiency solutions that work with, rather than against, practices in everyday settings?
40	What are the user profiles (time-use and electricity use) of energy ‘efficient’ appliances in real life; what rebound effects or unintended consequences are associated with these; and how can evidence on these inform better governance?
41	How is thermal comfort perception related to physiological, psychological and social influences; and how could understanding of these relationships help to improve energy efficiency and reduce energy consumption in everyday life?
42	What insights do the Humanities provide about how to create ‘cultures of energy efficiency’ that go beyond the usual dominant focus on consumer choices and ethical concerns?
43	What are the conditions that facilitate the acceptance and pursuit of energy sufficiency (e.g., living in smaller spaces, avoiding mobility, reducing consumption) over energy efficiency; and how can these conditions be scaled-up across society?
44	How are energy efficiency and sufficiency affected by changes to everyday life through ongoing processes of digitalisation (including, e.g., smart technologies, artificial intelligence and big data); and how do digital tools designed to improve energy efficiency and sufficiency interact with everyday practices?

Questions in this theme build on recent calls within SSH literatures for policy to move away from treating consumers as ‘Resource Men’, i.e., rational individuals that deliberately manage their energy consumption (Strengers, 2014), and to recognise that energy use and management is deeply linked with people’s context-specific experiences and understandings (Foulds et al., 2017; Verkade and Höffken, 2017). Several questions explicitly refer to practices, responding to lively debates in the literature around ‘social practice theory’ and how it can be operationalised and applied within energy policy (Breadsell et al., 2019; Hampton and Adams, 2018; Watson et al., 2020). In their focus on the interactions between technologies and practices, some questions advance thinking on the role of material infrastructures in constituting energy demand (Hansen et al., 2018; Ornetzeder et al., 2016; Shove and Trentmann, 2019). Other questions centre on experiential and cultural aspects of energy efficiency interventions; for example, experiences around thermal comfort (Madsen and Gram-Hanssen, 2017; Sahakian et al., 2021). However, a recurring idea within this theme is that energy use at the household level is inseparable from wider sociotechnical configurations, with questions addressing the relationships between systems of provision and the dynamics of everyday life. These questions resonate with emerging SSH research that engages with new modes of energy provision (Britton et al., 2021), shifting social rhythms (Blue et al., 2020), and the impacts of sociotechnical shifts like digitalisation (Morley et al., 2018).

This theme centres on the idea that energy facilitates all kinds of everyday practices, and that these practices are socially shared; a recognition that raises important questions for any research (within SSH or within STEM-centred projects) that aims to support the adoption, and long-term maintenance, of energy efficient or energy sufficient ways of life.

### Theme 4: Framing, defining and measuring energy efficiency

This theme centres on fundamental issues of how energy efficiency is defined, modelled, understood and measured (Table 4). The aim of this theme is to recognise that energy efficiency can be approached in many different ways, and to investigate how and why perspectives vary between actors and over time. SSH has questioned the persuasive power of models, measurements or numerical forecasts by unpacking, for instance: how these facts are produced; which decisions are made about what to include and what to exclude; and how political, cultural and social conditions frame what is accepted as plausible assumptions to be fed into the calculations (Dunlop, 2019; Hulme, 2011). Indeed, while energy efficiency is commonly defined as the relation between energy input and the work performed by energy; in an SSH context, the ‘work’ performed by energy is always already part of social, political, and cultural processes. In this way, SSH research, as demonstrated by the questions in this theme, draws attention to how the very concept of energy efficiency is subject to historical shifts and socially and culturally differentiated ideas about what energy should be used for.

**Table 4:** Theme 4 Social Sciences and Humanities (SSH) priority questions.

Question no.	Agreed SSH priority question
45	How are benefits and costs of EU, national and regional energy efficiency policies measured; and how can environmental and social outcomes, and unintended consequences, be more effectively included in these assessments (e.g., impact assessments for Directives)?
46	How is the making of energy efficiency policies influenced by forecasts, models, imaginaries and visions of energy supply and energy demand?
47	How have understandings of energy efficiency changed over time across different countries; and how have these visions affected technological pathways and lock-ins?
48	How are energy efficiency concepts used and implemented by policy(makers); and how can Social Sciences and Humanities insights improve this usage?
49	How do political and institutional contexts shape the ways in which energy efficiency is defined and measured; and how do these contexts determine who has authority in these processes of classification and quantification?
50	How do framings of energy efficiency vary between different social actors, including policymakers, industry, system operators, intermediaries, and energy service users; and how do these affect motivations for pursuing energy efficiency investments?
51	What values, assumptions and ethical choices are involved in the definition and measurement of energy efficiency; and what insights can the Humanities bring to understanding of these issues?
52	What responsibility do policymakers and energy efficiency ‘experts’ have to make indicators, sub-indicators and benchmarks (and related processes of creating these) transparent; and how could they be more transparent?
53	What are the taboos of energy efficiency (policy); and what energy efficiency issues remain unspoken due to inconvenience for those who benefit from the status quo (e.g., wealthiest, incumbents, particular disciplines, trade unions, other vested interests)?
54	How have neoliberalism’s tenets contributed to an emphasis on behavioural psychological and microeconomic framings of energy efficiency; and how might sociotechnical, cultural, structural and macroeconomic perspectives inform more fundamental challenges to current levels of energy demand?
55	To what extent might the pursuit of energy efficiency serve to reproduce unsustainable patterns of practice; and how can ‘energy efficiency’ narratives be redefined to encompass more systemic transformations?
56	How may ‘energy efficiency’ need to be redefined to adequately account for system- and sector-scale energy efficiency, rather than device-scale energy efficiency; and what are the implications of this redefinition for the forms of transformative change being pursued?
57	How can insights from social practice theories provide alternative understandings of energy efficiency; and how could re-organisation of energy-using practices contribute to greater energy efficiency and sufficiency at a societal scale?
58	How does the concept of energy sufficiency help to (radically) enrich and/or challenge current energy efficiency policies and understandings; and how can sufficient energy services and basic energy needs be defined?
59	In what ways has the term ‘user’ been implicitly and explicitly conceptualised across the Social Sciences and Humanities literatures on energy efficiency; and what are the implications of utilising broader perspectives on alternative modes of ‘use’?

Matters pertaining to frames, definitions and measurements are significant in SSH because one’s point of departure and inherent reference points will shape the findings generated and the conclusions/recommendations that can be provided (Royston and Foulds, 2021; Sovacool et al., 2018). As such, this theme’s questions cover investigations of the ways in which definitions, models, understandings and measurements of energy efficiency are produced by powerful ‘experts’, as well as how related policy positions are shaped by actors’ perspectives and agendas, e.g., the desire to present ‘hero stories’ (Janda and Topouzi, 2015, p. 516) of their work. This could relate also to the evolution of scientific tools, for example through the prism of risk analysis and post-normal science (Funtowicz and Ravetz, 1992; Kastenhofer, 2011).

Indeed, this theme is especially interested in more research on the implications of dominant framings for policies and their outcomes, and to highlight critiques and alternative framings, including those based on systemic approaches and those emphasising energy sufficiency, which draw attention to the important difference between ‘improving energy efficiency’ and actually reducing energy demand (Wilhite and Norgard, 2004). The questions in this theme thus suggest valuable insights to any project (SSH or STEM-based) that involves measuring and monitoring energy efficiency, or that engages with stakeholders who play a role in governing energy efficiency.

### Theme 5: Governance, policy and political issues around energy efficiency

The literature has called for a “more comprehensive governance framework” for energy efficiency (Dunlop, 2019, p. 6). Towards this end, more empirical analyses are required to identify what kind of governance is needed when implementing energy efficiency policies at different scales (Gupta and Ivanova, 2009; Jollands and Ellis, 2009; Pierre, 2000). Concurrently, a better understanding of the role of the relevant actors and institutions within the energy system is needed, as it can provide a deeper understanding of governance processes around energy efficiency, and their challenges (Delina, 2012; Pereira and da Silva, 2017). To give an example, we still know little about how the professional socialisation and the mindsets of civil servants at different levels of government affect the processes of agenda-setting, policy formulation, and implementation in the field of energy efficiency and sufficiency. In aligning with such arguments, this theme centres on how energy efficiency is governed by various actors and institutions across multiple scales (e.g., national and transnational). At the same time, a core aim of this theme is to critically examine the power dynamics at play, and to explore policy and governance issues around the emerging concept of energy sufficiency (Table 5). Towards this end, this theme directly responds to calls in the literature to expand on the concept of energy sufficiency (Burke, 2020; Herring, 2006; Shove, 2018) and to explore the potential synergies and complementarity between energy efficiency and energy sufficiency (Calwell, 2010; Thomas et al., 2015).

**Table 5:** Theme 5 Social Sciences and Humanities (SSH) priority questions.

Question no.	Agreed SSH priority question
60	What role can policy instruments play in advancing energy efficiency and sufficiency in different fields, such as ‘deep renovation’ of buildings, product policy, or digital infrastructures; and how do existing policy instruments perform on efficiency and actual energy savings?
61	What can be learned through a cross-national comparison of energy efficiency policies; how does best practice in energy efficiency policy diffuse between countries, regions and cities; and how can the underlying learning processes be facilitated?
62	What can be learned from a ‘policy mix’ analytical perspective on energy efficiency; specifically regarding policies’ coherence, consistency, development over time, and overall effectiveness; and how should policy mixes be designed to be most effective?
63	How (and to what extent) do the EU, national governments, and their associated regions and municipalities, coordinate policy decisions on energy efficiency; and how do they attempt to align these with spatial planning, environmental, social, and/or economic policies?
64	How has the mind-set and work of EU and Member States’ civil servants evolved, in response to the EU’s Energy Efficiency First principle that requires them to include energy efficiency gains in mainstream policy planning; and what is their influence on energy efficiency policy?
65	What kinds of governance are needed (and at what spatial and temporal scales) to support a move from energy efficiency projects as largely ad-hoc and piecemeal activities, into strategic and systemic programmes that transform the built environment and ensure an integrated focus on energy, water, waste and resource use in the long-term?
66	What is the role and responsibility of the state in managing the shift toward energy efficiency; and what patterns and types of energy transition are developed under different governance regimes (e.g., market-led, state-led, or civil society-led)?
67	What are the under-explored ‘leverage points’ for policymakers to intervene in social and built environment systems to promote energy efficiency; in particular, which intermediary actors could be more effectively engaged (e.g., tradespeople and community leaders)?
68	What is the role of intermediary organisations in creating an ‘entrepreneurial ecosystem’; and what kinds of organisational ecosystem governance can help scale up energy efficiency innovations?
69	Which geo-political factors play important roles in facilitating international cooperation for enhancing energy efficiency policies?
70	How can political will for driving energy efficiency be measured and stimulated?
71	How do power relations and vested interests affect (and potentially obstruct) policymaking on energy efficiency; and how can existing patterns of dominance in this sector be challenged?
72	To what extent, if at all, may energy efficiency policies be used by incumbent actors to reinforce the marginalisation of niche sociotechnical innovations?
73	How can the concept of sufficiency be effectively integrated into energy efficiency government policies, energy scenarios and anticipatory governance approaches; and how can energy sufficiency be ‘mainstreamed’ into other policies?
74	How does the political feasibility of energy efficiency policies compare to that of energy sufficiency policies; and to what extent are different countries adopting policies within each of these two paradigms?

This theme also focuses on how policies are designed and implemented, and the politics of energy efficiency. With different policies and policy instruments at play, the literature has called for more research on how new policies can be integrated within existing policy systems, while ensuring that both sets of policies (i.e., old and new) continue to work effectively and that they can be aligned without one obstructing the other (Neelis et al., 2007; Ringel et al., 2016). Comprising a further aim of this theme, the questions: seek to investigate how energy efficiency (and sufficiency) is currently addressed through policies; and highlight challenges, opportunities and learnings for policy improvement, as well as the main obstacles that these policies face. Within this context, one of the suggested routes is the ‘policy mix’ perspective, which can provide in-depth insights for policymakers regarding the *“coherence, consistency, development over time, and overall effectiveness”* (Q62) of energy efficiency policies.

While this theme concerns core issues within political, administrative and organisational studies, these questions will also be of value to any technical research that aims to achieve policy impact, or that requires an understanding of how governance contexts shape the emergence and development of technical innovations and transitions. The Working Group notes that while many of these questions are deliberately formulated to be wide-ranging, there are also more specific questions tacitly embedded within them, regarding the causal mechanisms and influences through which these governance and policy processes operate and take effect.

### Theme 6: Roles of economic systems, supply chains and financial mechanisms in improving energy efficiency

This theme centres on the roles of various kinds of economic systems, financial mechanisms, markets and supply chains in relation to improving energy efficiency and energy sufficiency (Table 6). In addressing the need for further research on the gap between legislation and implementation of energy efficiency policies by companies and industry—especially in regard to the ‘Energy Efficiency First’ principle (Nabitz and Hirzel, 2019)—the questions seek to provide a better understanding of the underlying reasons for this *“implementation gap”* (Q78), while striving to remedy the effectiveness of the policies (e.g., re-designing energy audits). Concurrently, given the differences between and across (sub-)sectors, the literature has called for more analyses that examine and/or compare different sectors (Fresner et al., 2017; Kluczek and Olszewski, 2017; Lund et al., 2017; Zuberi et al., 2020). This theme further expands the scope of enquiry to the interaction of energy efficiency measures with other economic policies (e.g., fiscal and monetary), financial mechanisms (e.g., carbon pricing), as well as economic dimensions of disruptive events like COVID-19.

**Table 6:** Theme 6 Social Sciences and Humanities (SSH) priority questions.

Question no.	Agreed SSH priority question
75	How do energy efficiency measures interact with other policy frameworks and financial mechanisms affecting the business and industrial sectors, such as fiscal and monetary policies and carbon pricing?
76	What impacts do energy audits of companies (such as those required by the EU’s 2012 Energy Efficiency Directive) have on the actual implementation of energy efficiency measures by those companies; and how can the design of auditing processes be made more effective?
77	In what ways do financial priorities in business and industry conflict with or complement energy efficiency goals; and to what extent are businesses implementing the ‘Energy Efficiency First’ principle, which stipulates that energy efficiency investments must be prioritised when it is cost-effective to do so?
78	Given that a large proportion of intentions to invest in energy efficiency measures (in existing buildings) are never carried out or are substantially delayed, how can Social Sciences and Humanities improve understandings of this implementation gap?
79	How can energy efficiency policy benefit from an analysis of the transnational markets and global supply chains that underpin different energy efficiency technologies, going beyond national-level assessments?
80	Given that Global South households often rely on second-hand donated electrical goods from Europe, what are the implications for importing energy (in)efficiency and how can these be addressed?
81	How can innovation in energy efficiency be encouraged in the Global South, so that inefficient consumption ‘lock-ins’ can be avoided?
82	How can stimulus packages after rare-destructive events (e.g., COVID-19 outbreak) be designed to include energy efficiency; to what extent is it viable to promote energy efficiency investments as an anti-crisis measure; and what would be the macro-effects of such an approach?
83	In what ways (if any) do post-COVID-19 recovery plans account for energy efficiency; how does energy efficiency complement and/or clash with economic recovery; and how will economic recovery affect the ability to achieve the goal of improving energy efficiency in different countries?
84	What new models and mechanisms for sharing, trading and accounting for energy resources are emerging; and what might these socio-economic innovations mean for energy efficiency and energy sufficiency?
85	How can policy support development of an adequately skilled workforce to implement the innovations needed to fulfil the EU’s energy efficiency targets; in particular, digital innovations in the building and construction sector?
86	How could alternative economic systems (e.g., slow, local, time-rich, high-satisfaction economies) contribute to energy efficiency and energy sufficiency?

This theme includes two questions (Q80, Q81) specifically dealing with the Global South; this reflects the fact that, despite our focus on the Global North, this theme deals with economic systems that are fundamentally global. As such, the questions consider issues around supply chains that link the Global North and Global South, and how emerging economies might avoid the lock-ins experienced by more energy-intensive nations. We note that within existing SSH literatures there is a major gap in knowledge on energy efficiency in Global South contexts, resulting in a narrower understanding of the effectiveness of the energy efficiency policies in different settings (Aliu, 2020; Chakravarty and Roy, 2016). While we do not have scope here to remedy this gap, this theme redirects the focus of analysis towards *“transnational markets and global supply chains”* (Q79), in an effort to provide a more holistic overview of the effectiveness and/or implementation of energy efficiency policies across sites and scales. This theme also delves into global issues of consumption, production and their intersections, responding to calls from the literature for analyses that move beyond ‘simplistic’ understandings of how energy efficiency relates to consumption and production (Gillingham et al., 2016). While Q85 considers issues around employment and workforce skills specifically in an EU context, these are of course critical concerns for research across global contexts (Malik et al., 2021).

The aim of this theme is to investigate how economic systems, supply chains and financial mechanisms can be mobilised and/or reorganised in order to contribute to energy efficiency and sufficiency goals. In particular, this theme raises a range of important questions that have not yet been adequately addressed through the already extensive work on energy efficiency within Economics, Marketing, Business Management and related disciplines. The questions will provide important insights to any research relating to the economics of energy efficiency, and the roles of business and industry. This theme shows how critical-SSH can open up radically new perspectives on socio-economic innovations and transitions, including transformations towards energy sufficiency.

### Theme 7: The interactions, unintended consequences and rebound effects of energy efficiency interventions

This theme concerns the ways in which energy efficiency interventions overlap and intersect with other areas of policy and practice (Table 7). As such, it draws on key strengths of energy-SSH; their attention to how specific technologies and interventions are embedded in wider social contexts and systems, and, related to this, their recognition of complex relationships across different sectors, often demanding interdisciplinary approaches. Questions in this theme challenge conventional techno-centric thinking about causation within energy efficiency interventions, by exploring a range of interactions and consequences that are often neglected or deemed ‘out-of-scope’.

**Table 7:** Theme 7 Social Sciences and Humanities (SSH) priority questions.

Question no.	Agreed SSH priority question
87	How can transdisciplinary approaches provide more nuanced understandings of ‘rebound effects’ of energy efficiency interventions (including effects on social practices, and on cultural and organisational dynamics); and how can such approaches inform more effective new policies and measures?
88	How can various ‘rebound effects’ or unintended consequences resulting from increasing energy efficiency be minimised through technological design, new policies, alignment with particular contextual conditions, or even the formulation of alternative approaches to reducing energy demand?
89	How does energy efficiency interact with other policy areas, such as urban planning, trade, gender, finance, labour policies, etc.; and in what ways can the promotion of other policy agendas conflict with energy efficiency goals?
90	How does transformation in various sociotechnical systems (e.g., housing, transport, agriculture, education, finance, etc.) affect change in the energy system; and what are the implications for the alternative framings of energy efficiency?
91	What is the degree of consistency between energy efficiency policies, energy market policies, environmental policies, welfare policies, economic and financial policies, across different countries; and how should this consistency be defined and measured?
92	How can energy efficiency policymaking and other environmental policymaking (regarding, e.g., climate adaptation, circular economy) be linked to create synergies for climate protection; and how can such approaches be mainstreamed?
93	How do new energy services and accompanying ICT platforms contribute to energy efficiency at societal scales; and what are the implications for inequalities; and how can policy address these?
94	How can Social Sciences and Humanities contribute to better qualifying and quantifying the non-energy-related benefits of energy efficiency; and how can this be translated into better Monitoring and Evaluation tools for policymakers?
95	How can energy efficiency objectives be aligned with public health objectives; for example, how can new packaging designs respect both public health and safety and energy efficiency aims?
96	What are the relationships between energy efficiency and healthy and productive indoor environments; and how can human-building interactions be improved to optimise all these outcomes?
97	What are the relationships between energy efficiency, energy demand and human well-being; and what roles could energy efficiency and energy sufficiency play in policy interventions to tackle inequalities in well-being?
98	What are the savings potentials of energy sufficiency initiatives across different (interconnected) sectors; and what are the suitable tools and possible business models to tap these potentials?
99	How do different forms of maintenance—for example, processes for monitoring, repairing and upgrading infrastructures—shape energy efficiency outcomes over long timescales?
100	How do different actors perceive and understand the interactions between energy efficiency and other policy agendas?

Some questions in this theme draw on the idea of ‘rebound effects’ (i.e., when efficiency gains lead to more energy use instead of less), which has been developed within techno-economic approaches to efficiency. However, this theme goes beyond these conventional perspectives to ask novel questions about how such effects are situated within social, cultural, organisational and policy contexts (as called for by Sonnberger and Gross (2018)). A particularly important contribution of SSH research concerns the diverse interactions between energy efficiency and other policy areas. This theme therefore explores how energy efficiency is embedded in a network of related agendas—be it on the level of individuals, households, institutions or national policies—and how energy efficiency (and sufficiency) interventions have complex ramifications. Here, this theme speaks to an emerging research agenda around ‘invisible energy policies’; i.e., policies that affect energy systems, but are not usually seen as energy policies (Butler et al., 2018; Foden et al., 2019; Jeliazkova et al., 2020; Royston et al., 2018). Several questions also explicitly focus on the complex, but vitally important, intersections between energy efficiency and health and well-being, as advocated by Ortiz et al. (2017). Additionally, the focus on diverse interventions and ‘invisible energy policies’ could be very helpful for early warnings in avoiding energy crises (Coyle and Simmons, 2014).

By exploring these various linkages, which are rarely recognised by researchers or policymakers, this theme aims to improve the evidence that informs energy efficiency policy, thus helping to avoid unintended consequences and to also optimise potential win-win scenarios or double-dividends. These questions thus have direct value to any research that aims to fully understand and effectively manage both the direct and indirect outcomes of energy efficiency policies and interventions, including work grounded in STEM disciplines.

## Concluding discussion

### The agenda: what do our 100 questions offer and how may they best be used?

The aim of this paper was to produce a research agenda outlining future SSH research priorities for energy efficiency in Global North contexts. We undertook a Horizon Scanning exercise that involved input from 152 energy-SSH researchers, who spanned 23 EU and Associated countries, 26 nationalities, 62 SSH (sub-)disciplines, both academic and industry organisations, and a range of career stages. A Working Group of 27 members—all of whom held SSH expertise in energy efficiency—used this wider input to select and edit 100 SSH priority questions, across seven inductively produced themes: (1) Citizenship, engagement and knowledge exchange in relation to energy efficiency; (2) Energy efficiency in relation to equity, justice, poverty and vulnerability; (3) Energy efficiency in relation to everyday life and practices of energy consumption and production; (4) Framing, defining and measuring energy efficiency; (5) Governance, policy and political issues around energy efficiency; (6) Roles of economic systems, supply chains and financial mechanisms in improving energy efficiency; and (7) The interactions, unintended consequences and rebound effects of energy efficiency interventions.

Taken together, these questions and themes (our ‘agenda’) represent beyond state-of-the-art research opportunities at the frontiers of SSH thinking on energy efficiency, illustrating how interdisciplinary synergies can be forged across SSH. The agenda highlights strategic opportunities for both researchers and decision-makers, especially given the fact that SSH receives significantly less energy/climate research funding than the Technical/Natural Sciences. We prioritised different perspectives, intentionally opening up the Horizon Scanning exercise as much and as early as possible. This fundamentally enabled a more inclusive approach and the creation of a truly interdisciplinary agenda, based on cross-disciplinary deliberative negotiations. Implicit to our agenda is the demonstration that interdisciplinarity need not be limited to bridging the Technical/Natural Sciences and the SSH; there is considerable interdisciplinary potential within the SSH, which is usually overlooked by policy and funding actors. Indeed, our research agenda showcases the possibilities afforded by embracing SSH problem definitions and lines of enquiry. It also, in the most basic sense, provides a list of ready-made research questions, which we hope may be especially helpful for early-career researchers and/or those newer to the energy-SSH field. Colleagues are welcome to use these questions to kick-start their own project proposals, studies and papers.

We understand that these questions are not representative of every SSH discipline/community and all its various members. We also appreciate that our agenda only represents views and preferences during that particular period in time. For example, should the exercise be conducted in 2022, we may expect more questions on the geo-politics and international governance challenges of energy efficiency, given the emerging effects of Russia’s invasion of Ukraine. However, it was never our intention to provide a definitive, long-lasting and representative agenda. Instead, our hope is that our agenda will spark debate and discussion, as indeed any good Horizon Scanning exercise should aim to do. We especially welcome responses from those who would advocate for different sets of research questions, based on different ontologies and epistemic points of departure.

### Points of departure: what do our 100 questions indicate about the directions of Social Sciences and Humanities (SSH) research on energy efficiency?

We begin here by reflecting on what we believe these 100 questions, together, are working against. We argue that the questions selected are a product of the SSH research communities’ representatives fighting back (consciously or not) against a mainstream paradigm that has long-dominated research and policy approaches to energy efficiency (and low-carbon energy transitions more broadly). In this way, what we now immediately discuss is actually represented in the agenda by its absence in the most part.

Mainstream energy efficiency research and policy approaches are often based on principles of (1) substitution of technological and energy inputs and of (2) modification of individual behaviours. These approaches are mostly dichotomous, as they split energy demand and supply. They either assume that technological and energy inputs (i.e., supply) can be changed without altering outputs (i.e., demand), or that individual behaviours (i.e., demand) can be modified without altering the material and social contexts that shape supply and demand. This dichotomy reflects a wider imagined separation between subject and object that societies have inherited from modernity and that still mostly informs the organisation of human activities in all sectors. This dichotomy is at the core of the separation that has been created between, for example, ‘hard’ and ‘soft’ sciences, or between producers and consumers. This dichotomy also explains how mainstream energy efficiency approaches can still be mostly linear and top-down, and do not consider how, for example, demand and supply actually co-evolve, or how technologies shape societies and vice-versa, etc. Such a point of departure is broadly absent from our questions.

In contrast, against this mainstream backdrop, our 100 questions regularly emphasise the importance of interconnectedness and complexity, as shown by “systems” being so commonly referred to, for instance. Similarly, the questions move far beyond out-dated overly linear, individualistic ontologies, with regular reference made instead to “communities” and “institutions”, for example. In addition, by so overtly distancing itself from technological determinism, this agenda also sought to recast and reframe notions of ‘innovation’ as a purely technological endeavour, to a societally driven and enabled endeavour (i.e., “social innovation”). Such a shift is essential if—again, as this agenda makes firmly clear—it is important to appreciate how changes induced in social practices by energy efficiency improvements always arrive in unexpected ways; and thus, that a key part of SSH’s remit should be to understand the lived experiences of energy efficiency, so that expectations can be better managed and detrimental impacts effectively minimised.

### Utility of approach: what do the processes and procedures underlying our 100 questions demonstrate to the field?

Our agenda, and its underlying Horizon Scanning exercise, emphasise the need for a pragmatic stance and coalition that enables the space to challenge, but still contains numerous normative touch points with, current policy pathways. This is well demonstrated by us producing a SSH Horizon Scan on energy efficiency, which is in itself a concept traditionally re-produced and enabled by technological determinism (e.g., European Commission, 2018). Indeed, the SSH literature would undoubtedly have organised recommendations in ways that cut through siloed, technical interests. The lead author’s experiences of seeking to influence energy-SSH funders (namely, within European Union institutions) certainly reiterate the futility of seeking to overthrow current funding system priorities, especially in the short-term.

This paper reiterates the importance of being more sensitive to different geographies, cultures and perspectives, in exploring, for example, how energy efficiency interventions should be better understood, designed, implemented and evaluated. Even within the confines of our Global North scope, both the existing literature and forward-looking themes have highlighted the importance of understanding and engaging with diverse and differentiated settings, actors and institutions, as well as attending to the intersections between different sites and scales; for example, through global supply chains for energy efficient technologies. Meanwhile, further Horizon Scanning work is urgently needed to bring Global South perspectives into the development of energy-SSH agendas. Across contexts, researchers would do well to (1) remember how energy policymaking cannot (and should not) be easily disentangled from energy efficiency (or, e.g., renewables integration) policymaking, and (2) better acknowledge the usually overlooked interconnections between energy (efficiency) policy and, for example, government finance, geo-politics and other policy areas. Energy efficiency research and innovation systems would indeed benefit by broadening its scope to include interactions and investigations of wider public policy programmes, as our final Theme 7 particularly emphasises. This agenda both highlights the complexities and the parameters involved in following such a pathway, as well as provides possible solutions by building on decades-long SSH-knowledge.

This paper has also demonstrated the utility of using Horizon Scanning methods to construct research agendas within/across SSH disciplines. We have applied methods traditionally used in the interdisciplinary Environmental Sciences, to SSH perspectives on energy efficiency, adapting them to better account for diversity within the field and with a view to greater methodological transparency than is typical of other published examples in the field. We assert such methods have considerable unfulfilled potential in the energy-SSH and welcome responses to these outcomes from colleagues.

## Data Availability

The source Horizon Scanning survey data have been made available in the FigShare repository, by the Energy-SHIFTS Consortium (2021). 10.25411/aru.17214221

## Footnotes

1 In these instances, it seems unlikely that a systematic deliberative methodology would have been employed, without any mention of such an approach.

2 This Horizon Scanning exercise was part of the EU Horizon 2020 funded project, Energy-SHIFTS, which reported directly to the European Commission’s Directorate-General for Research and Innovation (DG RTD). Part of this project’s remit was to provide direct advice and support to the European Commission on the directions of travel for SSH in EU Horizon 2020’s Secure, Clean and Efficient Energy Work Programme and in EU Horizon Europe’s Cluster 5 on Climate, Energy and Mobility. For more information, see: www.energy-shifts.eu. In the context of this paper, DG RTD was keen for SSH advice specifically on energy efficiency (rather than, e.g., using energy sufficiency as the foundational starting point) because of its ongoing commitments to fund research and innovation on energy efficiency (via e.g., EU SET-Plan).

3 Note that this Committee also kept fieldnotes throughout the whole Horizon Scanning exercise; these data were not included in this paper’s analysis, but did form the basis of a wider evaluation of the exercise itself (Bharucha et al., 2021b).

4 10 of the Working Group members were also interviewed by the Steering Committee, regarding past SSH debates on energy efficiency. While useful context for our Horizon Scanning, these data were not included in this paper’s analysis.

5 An European Commission list of Horizon 2020 Associated Countries is available: https://ec.europa.eu/research/participants/data/ref/h2020/grants_manual/hi/3cpart/h2020-hi-list-ac_en.pdf

6 European regions were classified using the UN’s Geographic Regions classifications for Europe’s regions (https://unstats.un.org/unsd/methodology/m49/). For those Horizon 2020 Associated Countries, which fell outside of UN European regional classifications, they were classified/counted in accordance with their nearest neighbouring European country.

7 Full guiding definitions of frontrunners and field leaders are available elsewhere (Foulds et al., 2019a, p. 18). Essentially: frontrunners are working at the boundaries of conventional academic structures, through their research’s interdisciplinarity, practical applications, exploratory nature, etc. Whereas, field leaders represent key SSH projects/communities, with more expertise in theory than in practical application.

8 The 199 questions with a median of 4 were ranked according to the highest total scores (i.e., mean) that they received across all Working Group members. The top 45 questions could be easily selected (total scores ranging from 136 to 119), but those questions with evaluation scores totalling 118 straddled the inclusion/exclusion boundary. Therefore, for questions with a median of 4 that straddled the in/out boundary, the remaining spots were selected using the percentage of 5-scores. Specifically, from the 12 (median of 4) questions that scored a total of 118, the remaining four spots were selected because they were ranked more highly in terms of the percentage of 5-scores (‘definitely include’ scores). Specifically, the selected four had a range of 35.48-29.03% of 5-scores, compared to a range of 25.81-19.35% for the other 8 questions.

9 The three sister Horizon Scans covered: renewables (von Wirth et al., 2020); smart consumption (Robison et al., 2020); and transport and mobility (Ryghaug et al., 2020).
